# Multiple exciton generation induced enhancement of the photoresponse of pulsed-laser-ablation synthesized single-wall-carbon-nanotube/PbS-quantum-dots nanohybrids

**DOI:** 10.1038/srep20083

**Published:** 2016-02-02

**Authors:** Ibrahima Ka, Vincent Le Borgne, Kazunori Fujisawa, Takuya Hayashi, Yoong Ahm Kim, Morinobu Endo, Dongling Ma, My Ali El Khakani

**Affiliations:** 1 Institut National de la Recherche Scientifique, Centre-Énergie, Matériaux et Télécommunications, 1650, Blvd. Lionel–Boulet, Varennes, Qc, J3X-1S2, Canada; 2 Faculty of Engineering, Shinshu University, 4-17-1 Wakasato, Nagano, 380-8553, Japan; 3 School of Polymer Science and Engineering, Chonnam National University, 77 Yonggong-ro, Buk-gu, Gwangju, 500-757, South Korea

## Abstract

The pulsed laser deposition method was used to decorate appropriately single wall carbon nanotubes (SWCNTs) with PbS quantum dots (QDs), leading to the formation of a novel class of SWCNTs/PbS-QDs nanohybrids (NHs), without resorting to any ligand engineering and/or surface functionalization. The number of laser ablation pulses (N_Lp_) was used to control the average size of the PbS-QDs and their coverage on the SWCNTs’ surface. Photoconductive (PC) devices fabricated from these SWCNTs/PbS-QDs NHs have shown a significantly enhanced photoresponse, which is found to be PbS-QD size dependent. Wavelength-resolved photocurrent measurements revealed a strong photoconductivity of the NHs in the UV-visible region, which is shown to be due to multiple exciton generation (MEG) in the PbS-QDs. For the 6.5 nm-diameter PbS-QDs (with a bandgap (Eg) = 0.86 eV), the MEG contribution of the NHs based PC devices was shown to lead to a normalized internal quantum efficiency in excess of 300% for photon energies ≥4.5Eg. While the lowest MEG threshold in our NHs based PC devices is found to be of ~2.5Eg, the MEG efficiency reaches values as high as 0.9 ± 0.1.

The prospect of exploiting multiple exciton generation (MEG), expanding thus efficient solar light photoconversion over the UV to near infrared (NIR) spectral range, has led to an ever increasing interest in quantum dots (such as PbSe, and PbS) as light harvesters^1^,^2^,^3^,^4^,^5^,^6^. The MEG is the process in which, upon the absorption of a single high-energy photon, at least two electron-holes pairs (excitons) can be generated. The efficiency of this process and its contribution to the performance of optoelectronic devices rely on the capability to separate electron-hole pairs and their subsequent efficient collection. The main factor that hinders taking advantage of MEG in colloidal quantum dots (QDs) is the poor inter-QD conductivity due to the presence of ligands on their surface. The mainstream approach to overcome this issue is to proceed with the so-called ligand exchange processing where long capping insulating ligands are replaced by shorter ones. This ligand exchange has been shown to improve photocharge mobility, leading thereby to higher efficiency photovoltaic devices^4^,^7^,^8^. Indeed, occurrence of MEG has been recently evidenced in this type of QDs involving PbX (X = Se, S) and CdSe^9^,^10^,^11^,^12^. Colloidal PbS-QDs photoconductors with high internal photoconductive gain, measured under a large external bias, have been demonstrated to exhibit MEG in the UV region^10^. Very recently, successive treatments of PbSe-QDs film with 1,2-ethanedithiol (EDT) and hydrazine, in order to increase the film conductivity, have shown external quantum efficiency (EQE) surpassing 100% that was attributed to the MEG process^12^. Despite these important advances, the issues caused by the low electronic coupling between colloidal QDs have been identified as a limiting factor for the improvement that MEG can contribute in the power conversion efficiency (PCE) of solar cells.

A very promising approach to exploit MEG is the hybridization of QDs with one or two dimensional nanostructures (including carbon nanotubes, nanowires or graphene), to form the so-called nanohybrids (NHs)^13^,^14^,^15^,^16^. In those NH systems, the QDs are used as light absorbers and charge generators whereas the other component (*e.g.* carbon nanotubes) acts as efficient charge conveyors. Moreover, the key advantages of using carbon nanotubes lie in their unparalleled charge carrier mobility, extremely highly aspect ratio, and high optical transparency when formed into thin films. All these characteristics are highly desirable for efficient photocharges transport in the NHs. Conventional chemical approaches for combining single-wall carbon nanotubes (SWCNTs) and colloidal QDs face problems such as a poor control over QDs diameter, low surface coverage of SWCNTs and inefficient charge transfer between QDs and SWCNTs due to the presence of ligands. Ligand-free attachment of semiconductor QDs to SWCNTs would indeed solve the issue of charge transfer; however, only a few works have tackled this challenging issue. In fact, using an ultrasonication based process, ligand-free attachment of CdSe QDs to multi-wall carbon nanotubes (MWCNTs) has been achieved^17^. However, this approach cannot be used for SWCNTs because of the damage induced by such a vigorous ultrasonication treatment, which ends in shortening the SWCNTs and thus lowering their conductivity^18^. Non-chemical approaches that rely on the direct physical deposition of QDs, such as pulsed laser deposition (PLD) or atomic layer deposition, entirely circumvent the need of using ligands^19^,^20^,^21^,^22^,^23^. As we have recently demonstrated, PLD offers an interesting synthesis route of NHs by directly growing PbS-QDs onto SWCNTs’ surfaces with a direct atomic contact between both constituents, overcoming the ligand exchange process and the difficulty of QD size control^21^.

In this work, we report on the use of the PLD technique for the synthesis of SWCNTs/PbS-QDs NHs with controllable PbS-QDs size and their subsequent integration into photoconductive (PC) devices. The NHs based PC devices were found to exhibit a photoconduction response spanning from the NIR region to the UV. More interestingly, the quantum efficiency spectra of the NH-based devices are found to exhibit an intense peak in the UV region, whose intensity depends on the size of the PbS-QDs. The strong UV photoresponse of the SWCNTs/PbS-QDs NH devices is demonstrated to originate from the MEG process taking place in the PbS-QDs. This is the first experimental evidence of the occurrence of MEG in the NHs based photoconductive devices where the PbS-QDs are grown by a physical PLD approach.

## Results and Discussions

**Figure 1:**
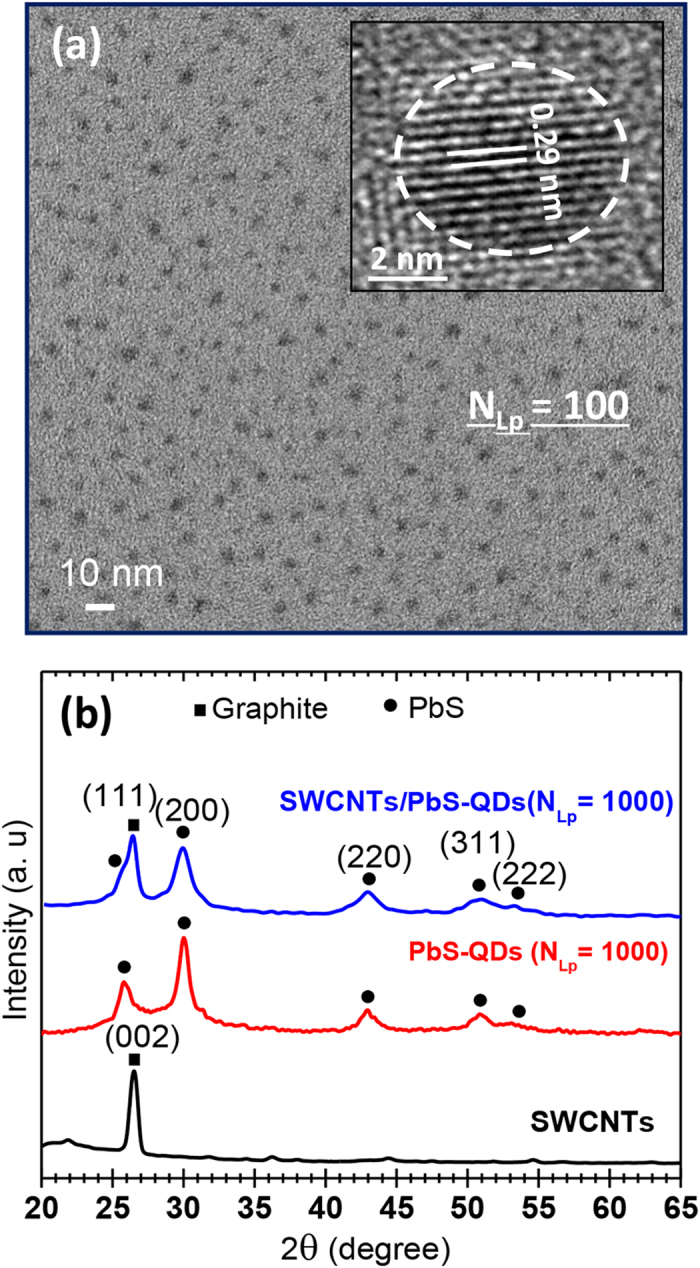
TEM and XRD measurements. (**a**) TEM image of isolated PbS-QDs PLD-deposited directly on a TEM grid at N_Lp_ = 100. (**b**) XRD spectra of the SWCNTs film, of the PbS-QDs (at N_Lp_ = 1000) and of the SWCNTs/PbS-QDs nanohybrid (at N_Lp_ = 1000) deposited on Si substrates.

We have synthesized PbS-QDs with different sizes by varying the number of laser pulses (N_Lp_) from 20 to 1000. Crystalline quality and QDs size were directly assessed through transmission electron microscopy (TEM) and X-ray diffraction (XRD). Fig. 1a shows a typical TEM image of the PbS-QDs directly grown on carbon-filmed TEM grid with N_Lp_ = 100. The PbS-QDs are seen to be well isolated with a relatively uniform size distribution centered around 4.2 nm. The inset of Fig. 1a shows the high resolution TEM image of an individual PbS-QD. It reveals the high degree of crystallinity of the QDs and the absence of any apparent stacking faults. Fig. 1b shows the XRD spectra of a SWCNTs film spray-coated onto Si substrate together with typical XRD spectra of PbS-QDs (at N_Lp_ = 1000) directly deposited on both SWCNTs and bare-Si substrates. The XRD spectra of the PbS-QDs on both substrates reveal its cubic phase with an fcc rocksalt structure (JCPDS 47-2123). The increased intensity of 2θ = 26^o^ peak in the PbS-QD/SWCNTs spectrum is due to the contribution from the graphite peak positioned at 26.5^o^. The latter is clearly seen to be present in the XRD spectrum of SWCNTs, confirming their graphite-like crystalline structure.

**Figure 2:**
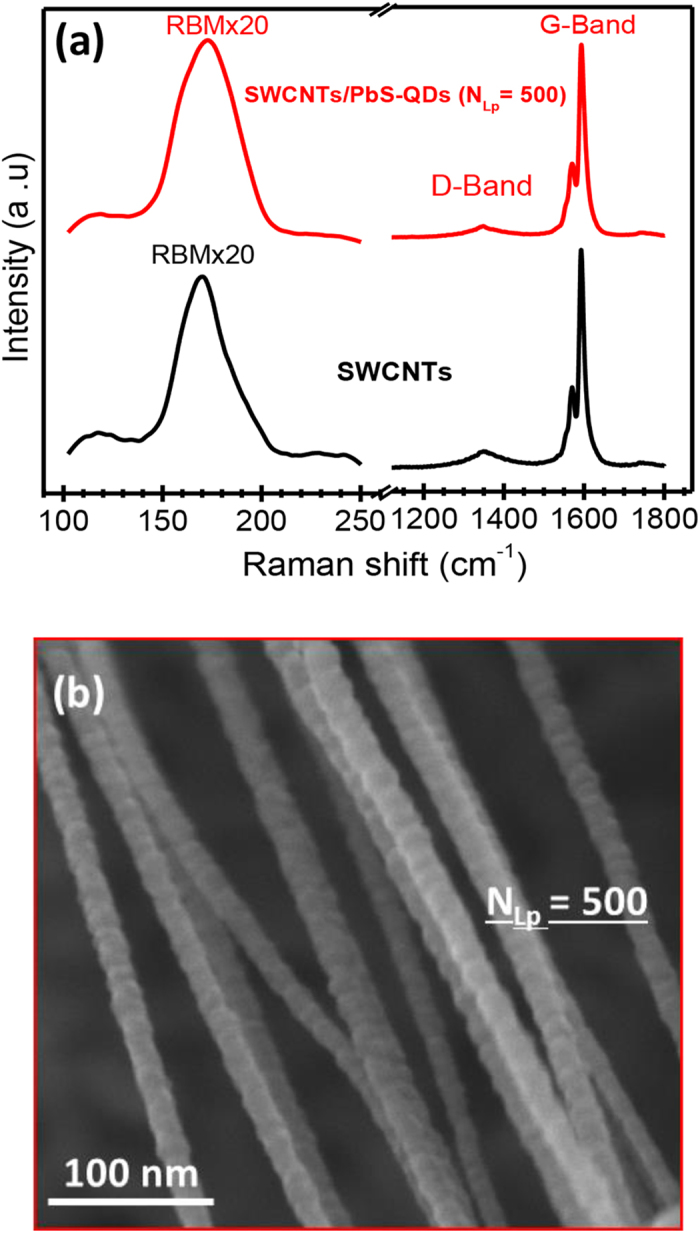
Raman spectra and SEM observation. (**a**) Raman spectra of the SWCNTs film and of the SWCNTs/PbS-QDs (at N_Lp_ = 500) NHs. (**b**) Typical SEM image of the NHs consisting of SWCNTs bundles completely covered by PbS-QDs (PLD-deposited at N_Lp_ = 500).

In order to investigate the effect of PbS-QDs decoration on structural characteristics of SWCNTs, Raman spectroscopy has been carried out by using a 514 nm laser as an excitation source. The typical Raman spectra before and after the PLD deposition of PbS-QDs on the SWCNTs are presented in Fig. 2a. This figure shows the typical fingerprint of SWCNTs consisting of the radial breathing mode (RBM) band (at 173.52 cm^−1^), the D-band (at 1349 cm^−1^) and the G-band (at 1592 cm^−1^). The similarity between the two spectra suggests that the presence of PbS-QDs on the surface of the SWCNTs does not seem to affect the SWCNTs electronic structure. The only noticeable change is the small widening of the RBM band and its shifting towards seemingly smaller nanotube diameters. This is thought to reflect the actual coating of the SWCNTs by PbS-QDs, limiting somehow their radial breathing. Finally, the low intensity ratio (∼0.1) of the D/G bands is indicative of the high degree of purity and good crystalline quality (no amorphous and/or disordered carbon) of our SWCNTs. After having clearly established the high-crystalline quality of the PbS-QDs and confirmed the preservation of structural properties of the SWCNTs (through the above-presented TEM, XRD and Raman spectroscopy characterizations), the efficiency of the PLD process to achieve conformal decoration of SWCNTs by PbS-QDs was made clear through scanning electron microscopy (SEM) observations. As an illustration, Fig. 2b shows a typical SEM image of the NHs (achieved at N_Lp_ = 500) where the SWCNTs’ surface is seen to be fully and conformally covered by the PbS-QDs.

**Figure 3:**
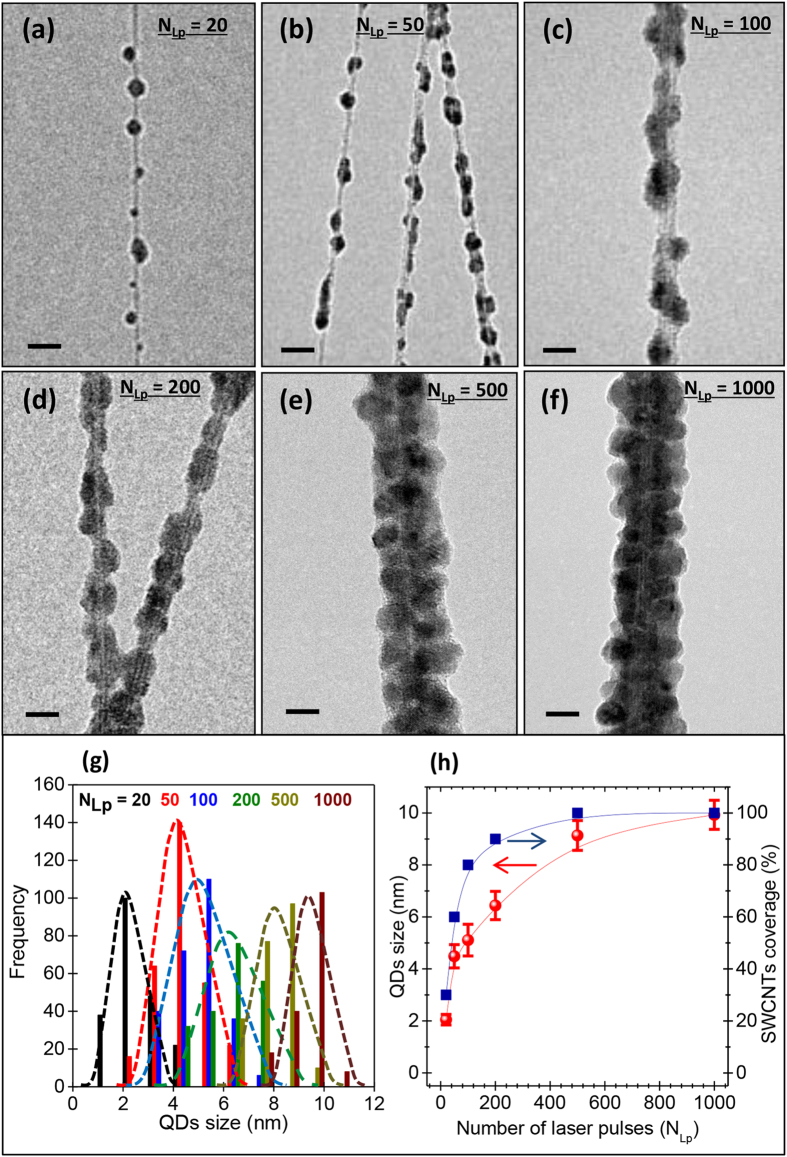
TEM imaging and size distributions of the PLD deposited PbS-QDs. (**a–f**) TEM images of SWCNTs/PbS-QDs nanohybrids made with N_Lp_ varying from 20 to 1000; (scale bar = 10 nm). (**g**) Histograms of size distributions of the QDs at different N_Lp_ values, as derived from TEM images. (**h**) Variation of the QD size and average surface coverage of the SWCNTs by the PbS-QDs, as a function of N_Lp_.

Through the variation of N_Lp_, NHs with different surface coverages of the SWCNTs were synthesized. Fig. 3(a–f) show the TEM images of NHs synthesized with increasing N_Lp_ values from 20 to 1000. At N_Lp_ = 20, isolated PbS-QDs are seen to attach at places on the surface of the SWCNTs bundle. The size and number of the PbS-QDs continue to increase until N_Lp_ = 500 where a full coverage of the SWCNTs’ surface is reached. In the histogram of Fig. 3(g), we show the size distribution of the QDs at different N_Lp_ values as measured from TEM images. At low N_Lp_ values (<100), isolated QDs attached on the SWCNTs with small diameters (varying from 2 to 5.3 nm) can be easily distinguished. For higher N_Lp_ values (≥200), the PbS-QDs grow more slowly in size and tend to saturate at a diameter of ∼10 nm at N_Lp_ = 1000. For N_Lp_ < 200, close observation of the QDs on the SWCNTs reveals their spherical shape, which evolves into a cubic one when the QDs grow enough (increasing N_Lp_) to impede their neighbor’s growth. The curves in Fig. 3(h) summarize the concomitant variations of the QDs size and associated surface coverage of the SWCNTs’ surface as a function of N_Lp_. It is worth recalling here that the PbS-QDs grow onto the surface of the SWCNTs. Indeed, the ablated species from the PbS target “land” onto the surface of SWCNTs, nucleate and start to grow as nanoparticles (or QDs). Some PbS-QDs embryos can also form in the plasma gas phase and be deposited directly on the substrate where they continue to grow from other “landing” ablated species. As the number of ablation laser pulses (N_LP_) is increased, not only the size of these QDs grows but also their density on the surface of nanotubes increases (as shown in Fig. 3(h)). At high N_LP_ values (≥500), a complete coverage of the SWCNTs by the PbS-QDs layer is reached (the SWCNTs are sort of wrapped into a PbS-QDs outer layer; as shown in Figs. 2(b) and 3(e)). As the SWCNTs are laterally lying down onto the quartz substrate, the PbS-QDs grow on their surface, which is their most exposed part to the laser ablation flux. Consequently, the chances to have some QDs possibly growing inside the tubes are very low to not to say nil. As a matter of fact, we have never seen any PbS-QD inside the SWCNTs despite the countless TEM observations.

**Figure 4:**
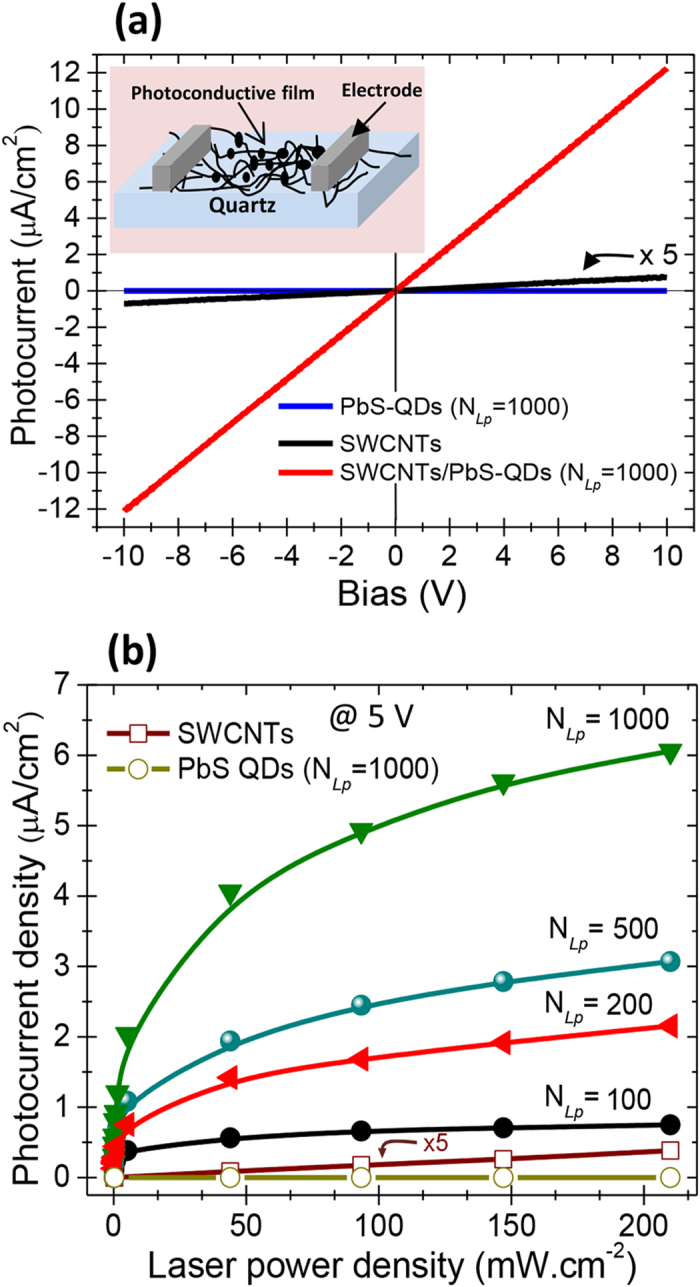
Photocurrent measurements. (**a**) Typical I_ph_-V curves of the PbS-QDs alone, the SWCNTs (without PbS-QDs decoration) and SWCNTs/PbS-QDs nanohybrids based photoconductive devices, under 633 nm laser illumination. (**b**) Photocurrent (at 5 V) of the SWCNTs/PbS-QDs nanohybrids devices made with different N_Lp_ (from 100 to 1000) as a function of the laser power density.

The SWCNTs/PbS-QDs NHs were integrated into the PC devices, of which basic architecture is sketched in the inset of Fig. 4a. Two reference devices have been fabricated by using a pure SWCNTs film (without PbS-QDs) and a pure PbS-QDs (N_Lp_ = 1000) film directly deposited onto quartz (without the SWCNTs underlying layer). The PC results reported here are representative of 3 to 5 devices, for a given N_LP_ condition, and their PC response is found to be quite reproducible within an experimental error margin of ≤4%. Figure 4a compares the I-V curves of SWCNTs and PbS-QDs reference devices to that of a typical SWCNTs/PbS-QDs NH device (achieved at N_Lp_ = 1000), under a 633 nm laser illumination with a light intensity of ~200 mW.cm^−2^. It is important to note that no photocurrent has been detected from the devices made only with the PbS-QDs (N_Lp_ = 1000). Indeed, the PbS-QDs deposited at N_Lp_ = 1000 onto a quartz substrate form a continuous film of ~30 nm thick (assuming an average deposition rate of 0.03 nm/laser pulse) consisting of PbS-QDs with an average diameter of ~10 nm. These PbS-QDs films are sufficiently resistive to hinder any charge flow through the devices at the applied voltages. On the other hand, the PC devices made solely by SWCNTs (without any PbS-QDs decoration), were also found to generate only a very weak photocurrent (Fig. 4a). In contrast, the association of the PbS-QDs with the SWCNTs to form the NHs has led to PC devices exhibiting a strong photocurrent, which is ~90 times larger than that produced from the SWCNTs device at a bias of 10 V. This demonstrates the complementary and synergistic roles played by both PbS-QDs and SWCNTs components of the NHs (i.e.; the photons are absorbed by the QDs and the generated photocharges are rapidly conveyed by the underlying high mobility SWCNTs).

For the active PC devices, the photocurrent density is seen to vary linearly with the applied bias.

By varying the laser illumination conditions, we were able to investigate the power dependence of the photocurrent of the NHs devices at a biasing voltage of 5 V. Figure 4b shows that regardless of the used N_Lp_, the photocurrent continuously increases with the laser power density. Nevertheless, one can notice that the efficiency of production of photocurrent is much higher at low laser power densities than for higher ones (≥100 mW.cm^−2^), for which the curve slopes of Fig. 4b are less steep. This suggests that photocharges recombination may occur in the entangled SWCNTs network particularly under high illumination conditions. In fact, at low power density deep trap states are available, allowing longer lifetime of electrons and lower recombination. In contrast, at high power densities, the deep trap states are filled, only short-lived trap states (shallow traps) are available, increasing thereby the recombination which affects the efficiency of production of photocurrent. Such a less efficient generation of photocurrent at high light intensities has been also observed by Konstantatos *et al*. and attributed to the recombination that increases with the laser power^24^,^25^.

On the other hand, for a given laser illumination condition, Fig. 4b shows that the photocurrent increases with N_Lp_ suggesting that the absorption of the photons by the PbS-QDs is driving the photocurrent generation process. In fact, the photons absorption in the PbS-QDs leads to the creation of excitons of which photocharges are rapidly transferred to the underlying SWCNTs. This has been inferred from the strong quenching of the photoluminescence (PL) of the PbS-QDs once associated with SWCNTs to form the nanohybrids (as we have demonstrated in a previous paper^21^). By using work function values of ~4.7 eV for PbS-QDs^26^,^27^ and ~5.1 eV for SWCNTs^28^,^29^ and assuming an average bandgap energy value of ~0.9 eV for both PbS-NPs and SWCNTs, the band diagram (depicted in Fig. 3d of ref. 21) confirms an energetically favorable charge transfer from PbS-QDs to SWCNTs. It is important to note that the average E_g_ value of 0.9 eV for PbS-QDs is quite realistic as it falls well within the E_g_ values (i.e.; 0.6–1.2 eV) of the PbS-QDs investigated in the present work. On the other hand, the E_g_ of SWCNTs is known to be inversely proportional to their diameter and the 0.9 eV value for ~1.3 nm diameter SWCNTs is quite representative for our SWCNTs (both Raman and HR-TEM observations have shown a predominance of SWCNTs with diameters in the (1.25–1.30) nm range^30^,^31^. This energetically favored charge transfer mechanism of the photocharges from PbS-QDs to SWCNTs along with the direct atomic contact between PbS-QDs and SWCNTs (through the formation of C-S bonds, as revealed by XPS analyses^32^) are at the origin of the fastest photoresponse^21^ exhibited by our NHs.

On the other hand, the bandgap (E_g_) values of the PbS-QDs were derived from their associated PL emission spectra (as the one illustrated in Figure S1 in the supporting information for ~4.2 nm-diameter PbS-QDs) for the different N_Lp_ values^33^. In a previous work, we have established a direct relation between the size of PLD PbS-QDs and their PL-determined bandgap^33^, in accordance with the theoretical model of Wang *et al*.^34^. The use of PL spectra to determine the E_g_ values of our PbS-QDs is quite convenient as their excitonic absorption peak can be hardly pinpointed form their UV-Vis spectra mainly because of the very nature of our PbS-QDs films. Indeed, unlike colloidal suspensions where the probed volume contains sufficient PbS-QDs to yield a significant absorption and thereby enable an easy observation of the excitonic absorption peaks, the probed amount of PbS-QDs in our PLD-deposited films is very tiny. For example, the very thin film of PbS-QDs deposited with N_Lp_ = 100 onto a quartz substrate, would have an equivalent thickness of only ~3 nm (assuming the average PLD deposition rate of ~0.03 nm/ablation pulse). In fact, this film is rather discontinuous and consists of sparse PbS-QDs having a diameter of ~4.2 nm. Figure S1 in the supporting information section shows both the absorbance and PL spectra of the ~4.2 nm-diam. PbS-QDs film deposited onto a quartz substrate.

**Figure 5:**
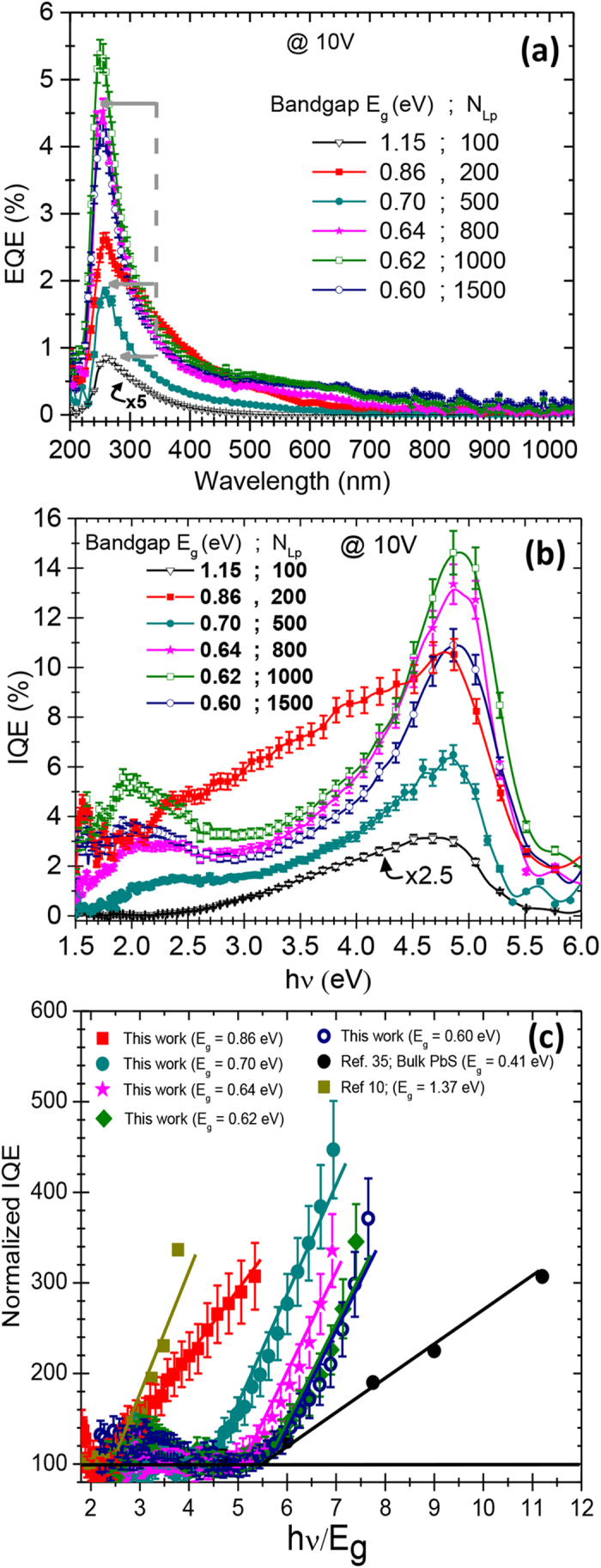
EQE and IQE spectra. (**a**) Photoconductive EQE spectra, measured at a biasing voltage of 10V, as a function of incident photon wavelength and (**b**) their corresponding IQE spectra as a function of the incident photon energy for the different NHs devices made with N_Lp_ varying from 100 to 1500. (**c**) Normalized IQE of the various SWCNTs/PbS-QDs devices made with different N_Lp_ values as a function of the photon energy/QD-bandgap (hν/Eg) ratio. For comparison purposes, data from literature are also included.

To investigate the effect of N_Lp_ on the photoresponse of the devices, spectrally resolved EQE spectra of the NHs based devices, which characterize the number of photogenerated electrons per incident photon at every wavelength (as explained in the Methods section), were systematically measured over all the UV to NIR range. Fig. 5a shows the EQE spectra of the NHs devices made with increasing N_Lp_ values from 100 to 1500. First, it is worth noting that the EQE photoresponse spectra of the SWCNTs/PbS-QDs nanohybrids are quite similar to the UV-Vis absorption spectra of either PbS-QDs alone or those of the SWCNTs/PbS-QDs nanohybrids (Figs S2 and S3 in the supporting information). In particular, the higher absorption of the PbS-QDs at UV frequencies translated into an EQE peak around 260 nm for the NHs based PC devices. This confirms that the photon absorption in the NHs is occurring mainly through the PbS-QDs. However, the EQE decrease observed for wavelengths shorter than 240 nm is rather intriguing, as one would expect the EQE to remain constant at its high value, in accordance with the absorption spectra of the SWCNTs/PbS-QDs. The physical reasons for such EQE decrease at shorter UV wavelengths remain unclear at the moment and one has to consider the EQE variation in the (200–240 nm) with caution. Nonetheless, for very short wavelengths, some stray light can lead to an overestimation of the light intensity coming from the EQE system’s monochromator, which would in turn decrease the calculated value of EQE. Also, one might speculate about the high absorption of the CNTs at shorter UV frequencies that could lead to a decrease of the photons available to PbS-QDs. Finally, an enhanced interfacial recombination of carriers at such high photon energies may also contribute to a drop in EQE, as this has been invoked in the case PbSe-QDs based solar cells^12^.

A close examination of the EQE spectra of all the devices reveals that all the devices exhibit a photoresponse peak in the (~250–270) nm UV range, depending on their N_Lp_ value. The height of this UV-response peak is found to depend on the N_Lp_, and, more interestingly, its intensity increases by steps for certain N_Lp_ values (gray arrows in Fig. 5a). Indeed, despite the differences in the PbS-QDs decoration conditions of the SWCNTs, NHs based devices made with N_Lp_ = 200 and 500 both produce a similar amount of photocurrent which is significantly higher than that produced by N_Lp_ = 100. Likewise, the devices made with N_Lp_ = 800, 1000 and 1500 produce almost the same photocurrent intensity (at 260 nm) which is more than twice higher than that of the N_Lp_ = 200–500 group. This clearly shows that the photocurrent generation does not increase linearly with N_Lp_ but seems to be rather increasing by steps following some thresholds. To further investigate the origin of the presence of the 3 different EQE intensity plateaus in Fig. 5a (i.e.; for N_Lp_ = 100, for N_Lp_ = 200–500, and N_Lp_ = 800–1500), the EQE spectra of the different NHs were divided by their respective optical absorption (A) spectra (shown in figure S3 in the supporting information). The resulting internal quantum efficiency (IQE) spectra, account for the number of photoelectrons generated by the devices per absorbed photon (rather than per incident one), at every wavelength. Figure 5b shows the obtained IQE values, as a function of the incident photon energy, for the SWCNTs/PbS-QDs based PC devices made with different N_Lp_ values. Accessing the IQE spectra is highly relevant to single out the photogeneration process due solely to photons absorption while getting rid of other contributions (mainly incident photons loss through reflections). By examining the IQE spectra of Fig. 5b, it appears that for all the devices, IQE remains more or less insensitive to hν in a sort of “flat” regime (at low photon energies) up to a certain threshold, and then prominently rises for higher photon energies. (As an example, for NHs devices made with N_Lp_ = 500 (E_g_ ~ 0.7 eV), even if there is a sort of bump around 2 eV, the IQE is seen to really take-off for photon energies >3.1 eV.) The averaged value around which the IQE fluctuates in the “flat” part of the spectra (low photon energies range) was found to be device dependent. To be able to compare all the devices (with different PbS-QD sizes), we have considered this “flat” part of every IQE spectrum as a sort of baseline. Thus, by dividing each IQE spectrum with its corresponding baseline value (i.e. averaged IQE values in the “flat” part of the IQE spectrum corresponding to low photon energies), we have defined what we refer to as a normalized IQE (NIQE) spectrum (as detailed in figure S4 and associated explanation in the supporting information). These NIQE spectra permit to single out the relative rise of the IQE of each device with respect to its associated baseline. Moreover, reporting these NIQE curves against the hν/Eg ratio enables direct comparison of different sizes of PbS-QDs with different bandgaps on the same plot. The NIQE spectra of our NHs based PC devices were plotted and compared with the rather scarce literature results available for either colloidal PbS-QDs and bulk PbS^10^,^35^. Interestingly, Fig. 5c shows a sort of a universal behavior where all the devices exhibited the same steep increase (with the same slope, as it will be discussed hereafter) in their NIQE after a certain photon energy threshold. The threshold value at which the photocurrent generation rise takes off is found to shift to higher photon energies as the PbS-QDs grow in size. Such a behavior along with the surprisingly higher IQE response at higher photon energies strongly suggests the occurrence of MEG in our NHs based PC devices. The occurrence of a photoconductive gain would have been a possible explanation for the photocurrent enhancement, as this has been invoked in the case of colloidal PbS-QDs, where photosensitization was associated with the presence of oxide species on the QDs’ surface^36^,^37^. Such photosensitization process involves preferential trapping of charge carriers in the PbS-QDs. If such photosensitization would be present in our PbS-QDs, it should not be depending on different photon energy thresholds for different QD sizes (as seen in the NIQE spectra of Fig. 5c). In sum, our results are more in line with the occurrence of MEG for at least the two following reasons: (i) the existence of a photon energy threshold beyond which the MEG occurs and (ii) higher NIQE values were exhibited by smaller PbS-QDs (as seen in Fig. 5c) because of higher quantum confinement. Both facts are typical of the occurrence of MEG effect^10^,^38^,^39^,^40^,^41^. In fact, the energy value (which is QD size dependent) beyond which the NIQE starts to increase linearly has been defined as the MEG threshold in previous reports^38^,^39^. For example, the NHs made with PbS-QDs at N_Lp_ = 200 (their E_g_ = 0.86 eV) are seen to yield a NIQE of ∼300% at hν = 4.5E_g_ while the NHs made with PbS-QDs at N_Lp_ = 500 reach only 150%, showing thereby that the MEG occurring in our NHs based PC devices is more efficient as the QDs size decreases (higher quantum confinement). These results are in agreement with the work of Midgett *et al*., where the MEG of both PbS and PbSe QDs was investigated as a function of size and composition of the QDs^42^. Here, for all the QDs and beyond their MEG threshold, the NIQE increases almost linearly with the same slope. Indeed, by using the same model as Semonin *et al*.^12^, we have found that the MEG efficiency (η_MEG_) in our NHs based PC devices, calculated from the slope of the linear fit (dash line in Fig. 5c) 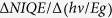, is of ∼0.9 ± 0.1. This value is higher than the highest value (of 0.42) reported by Midgett *et al*.^42^, for 4.2 nm QDs, indicating that the MEG from our PLD grown PbS-QDs is highly efficient. There are very few reports on MEG that have shown a η_MEG_ value as high as 0.9. For example, very highly efficient carrier multiplication of η_MEG_ ranging from 0.55 to 1 has been reported by Aerts *et al*.^43^ in PbS nanosheets with thicknesses from 7 to 4 nm. Moreover, it is important to note that this efficient MEG occurring in our PbS-QDs could not be evidenced from photocurrent measurements without their association with the SWCNTs to form the NHs. At this point, it is worth recalling that MEG has been also observed in CNTs but more particularly for some specific chiralities and in some cases at relatively low temperatures^44^,^45^. In the present work, our SWCNTs consist of a random mixture of different chiralities and the PC/EQE measurements were performed at room temperature, lowering thereby the chances to see any carrier multiplication contribution from the SWCNTs. As a matter of fact, the I-V curves (Fig. 4a) of the devices made solely with SWCNTs have shown a negligible photocurrent (~90 times lower than that yielded by SWCNTs/PbS-QDs nanohybrid based devices), ruling out the possibility of any contribution of SWCNTs to the carrier multiplication exhibited by the nanohybrids. On the other hand, the presence of SWCNTs (or other highly conductive network of nanowires) is definitely essential to collect and transport the photocharges. Indeed, PbS being very resistive, typical PbS-QDs photodetectors employ intricate electronics and high voltages to measure photocurrent. For example, an electric field as high as 2 V/μm has been applied by Sukhovatkin *et al*. in order to be able to demonstrate MEG from their colloidal PbS-QDs based photoconductive device^10^. Here, we show that the presence of SWCNTs has made it possible to measure wavelength resolved EQE spectra at a very low bias (0.002 V/μm in our case versus 2 V/μm in reference 10). Finally, the efficient collection of photocurrent in the NHs is favored because of the direct contact (without ligands or organic molecules) between the PbS-QDs and the SWCNTs, allowing a fast charge transfer.

In summary, the PLD technique was successfully used to achieve SWCNTs/PbS-QDs NHs made from the controlled decoration of SWCNTs by physically grown PbS-QDs. The size dependent bandgap of the PbS-QDs was varied and its effect on the optoelectronic response of the NHs investigated. We were thus able to show that the SWCNTs/PbS-QDs NHs exhibit strong UV-visible photoconductive response, which is found to be modulated by the size of the PbS-QDs. This strong enhancement of the photoconductance of the NHs devices is demonstrated to originate from MEG in our PbS-QDs. This is the first demonstration of MEG occurrence in photoconductive devices made of physically (PLD) processed SWCNTs/PbS-QDs NHs, where neither ligand engineering nor surface functionalization were used to attach the QDs to the nanotubes. The direct atomic contact between the QDs and the nanotubes together with the unique charge transport ability of the SWCNTs are key factors to reveal MEG in these NHs.

## Methods

### Synthesis and characterization of PbS-QDs and SWCNTs/PbS-QDs nanohybrids

We deposited the PbS-QDs by means of PLD onto various substrates, including SWCNTs films (for the synthesis of the SWCNTs/PbS-QDs NHs), quartz slides and carbon polymer coated TEM grids for different characterization purposes. In our PLD process, a KrF excimer laser (λ = 248 nm; pulse duration = 20 ns; repetition rate = 20 Hz) set at a power of 2.5 × 10^8^ W/cm^2^ is focused onto a pressed PbS powder target under helium background atmosphere (at a pressure of 500 mTorr). To achieve a uniform PbS-QDs deposition, the substrates are placed on a rotating substrate holder, which is parallel to the target and at a distance of 5 cm. The SWCNTs used in this work were also synthesized by means of the laser ablation approach where a UV laser is shone onto a catalyst-loaded graphite pellet under an Ar atmosphere. More details on the laser ablation synthesis of these SWCNTs and their related structural properties can be found elsewhere^30^,^31^. The PbS-QDs and the NHs samples obtained after coating the SWCNTs by the QDs were systematically characterized by X-ray diffraction with a Philips X’pert diffractometer using a Cu K α radiation source (λ) of 0.154 nm, and by field emission transmission electron microscopy (TEM), using a JEOL 2100F microscope. The optical absorption spectra of the samples were measured by means of a cary 5000 UV-vis-NIR spectrophotometer.

### Device fabrication

Purified SWCNTs suspended in a dimethylformamide (DMF) solution (0.1 mg.ml^−1^) are spray-coated on quartz substrates until a resistance of 1 MΩ is reached, ensuring percolation of the SWCNTs. Then, different laser ablation pulses (N_Lp_) values of PbS-QDs, in the (20–1500) range, were deposited directly on the SWCNTs. Finally, photoconductive devices based on the SWCNTs film decorated with PLD-deposited PbS-QDs were fabricated by depositing two parallel silver electrodes (0.5 cm apart) on the NHs films (as schematically illustrated in the inset of Fig. 4a).

### Photoconductive Device characterization

The current-voltage (I-V) curves of the NH based PC devices were collected with an HP-4140 under illumination of a 633 nm laser at different power intensities. The external quantum efficiency (EQE) of the PC devices is calculated as follows, 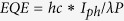 where P is the incident light power at a given wavelength (λ) and c is the speed of the light. The photocurrent (i.e.; the net current produced by the device under “light” and “dark”, at a given applied voltage is defined as I_ph_ = I_light_ - I_dark_) at each incident wavelength was measured by means of a lock-in amplifier (Ametek 1256). The incoming monochromatic light (chopped at frequency of 6 Hz) was focused to a spot size of 0.2 × 0.2 cm^2^ onto the device, which is biased by an applied voltage of typically 5 or 10 V. The incident light power was systematically measured at each wavelength with a calibrated photodiode (Newport 918D). Finally, as explained above, the IQE values of the different NH based PC devices were simply obtained by dividing their EQE value, at a given wavelength, by its corresponding absorbance (A) value at the same wavelength as follows, 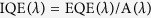.

## Additional Information

**How to cite this article**: Ka, I. *et al*. Multiple exciton generation induced enhancement of the photoresponse of pulsed-laser-ablation synthesized single-wall-carbon-nanotube/PbS-quantum-dots nanohybrids. *Sci. Rep.* **6**, 20083; doi: 10.1038/srep20083 (2016).

## Supplementary Material

Supplementary Information
