# Supplementary material for: Multiple exciton generation induced enhancement of the photoresponse of pulsed-laser-ablation synthesized single-wall-carbon-nanotube/PbS-quantum-dots nanohybrids
Source: Sci Rep. 2016 Feb 2;6:20083. doi: 10.1038/srep20083 (PMC4735719; doi:10.1038/srep20083)
Supplement: Supplementary Information [file srep20083-s1.pdf]

Supporting information

Multiple exciton generation induced enhancement of the photoresponse of pulsed-laser-ablation synthesized single-wall-carbon-nanotube/PbS-quantum-dots nanohybrids

Ibrahima Ka,[1] Vincent Le Borgne,[1] Kazunori Fujisawa,[2] Takuya Hayashi,[2] Yoong Ahm Kim,[2,3] Morinobu Endo,[2] Dongling Ma,[1] and My Ali El Khakani [1,]*

[1]Institut National de la Recherche Scientifique, Centre-Énergie, Matériaux et Télécommunications, 1650, Blvd. Lionel–Boulet, Varennes, Qc, J3X-1S2, Canada
[2]Faculty of Engineering, Shinshu University, 4-17-1 Wakasato, Nagano, 380-8553, Japan

[3]School of Polymer Science and Engineering, Chonnam National University, 77 Yonggong-ro, Buk-gu, Gwangju, 500-757, South Korea

***Corresponding Author:** Prof. My Ali El Khakani; E-mail: elkhakani@emt.inrs.ca

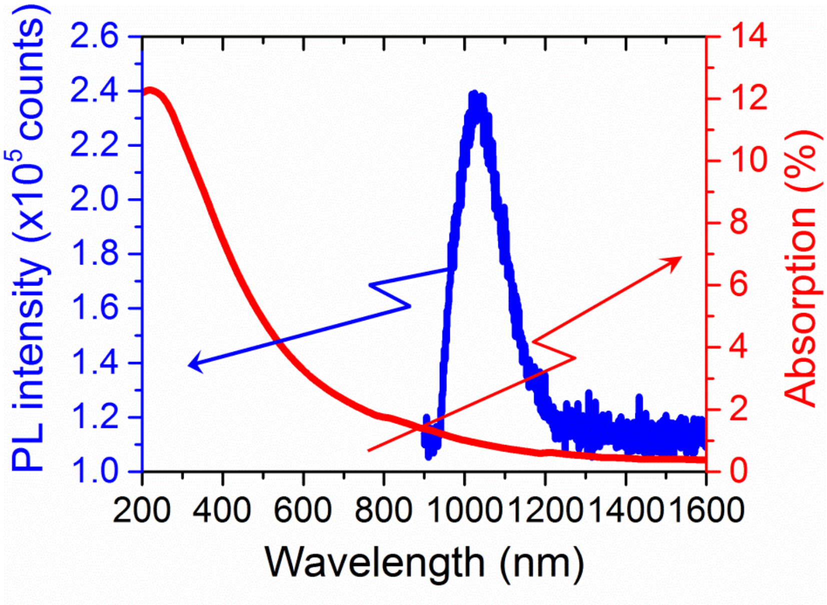

Figure S1. Absorption and photoluminescence spectra of PbS-QDs of size 4.2 nm made with N_{Lp} = 100 and deposited on quartz substrate.

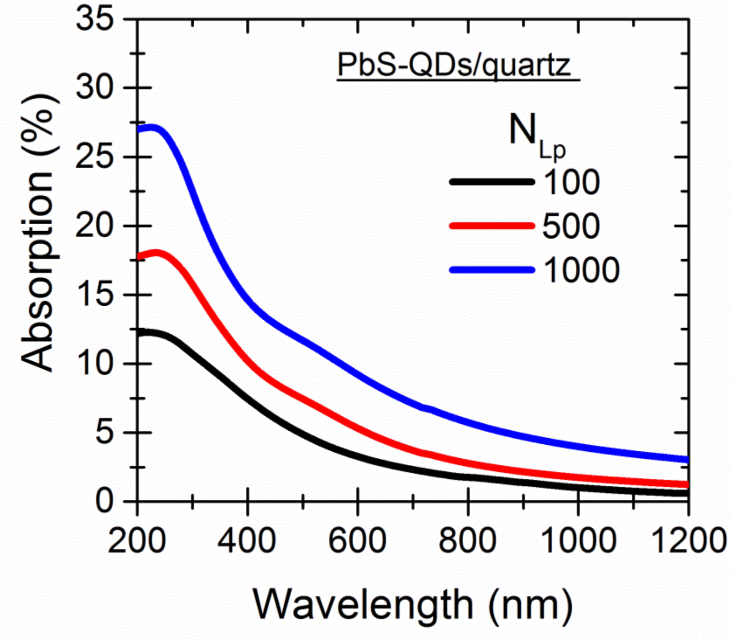

Figure S2. Absorption spectra of PbS-QDs made with N_{Lp} = 100, N_{Lp} = 500 and N_{Lp} = 1000.

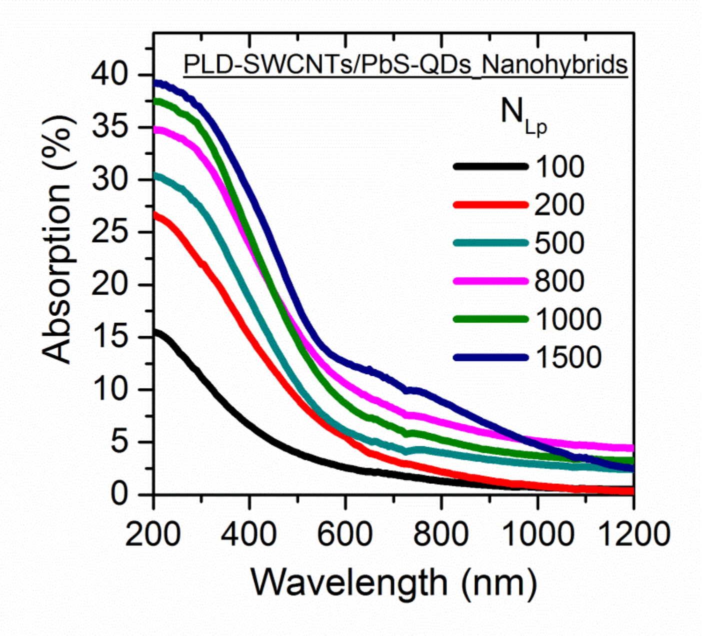

Figure S3. Absorption spectra of SWCNTs/PbS-QDs nanohybrids made with N_{Lp} varying from 100 to 1500.

As explained in the article, all the IQE spectra were normalized with respect to the average value of their low energy part (< 3 eV), which was considered as a sort of baseline. This enables the direct comparison of the various devices on the same scale. The same procedure has been previously used by others[1-5] in order to determine the photon energy MEG threshold and to evidence the occurrence of MEG. It is also worth recalling that the MEG threshold represents the photon energy from which the IQE starts increasing almost linearly. Figure S4 shows the different processing steps of the IQE spectrum (for the sample with Eg= 0.7 eV; N_{Lp} = 500) to obtain its corresponding NIQE curve. First, the initial IQE spectrum (Fig. S4(a)) is simply replotted by dividing the X axis by the corresponding Eg value (0.7 eV here) to express it in a unitless quantity (i.e.; hν/Eg), as shown in Fig. S4(b). In this figure, we identify the average value of the baseline in the low energy part before the almost linear increase of the IQE (blue dashed-line), which is found here to be of about 1.45. Finally, each IQE value in the flat and linearly increasing parts (inside the red square) is divided by the “baseline average value of 1.45” and multiplied by 100 to obtain the NIQE curve (as shown here in Fig S4(c)).

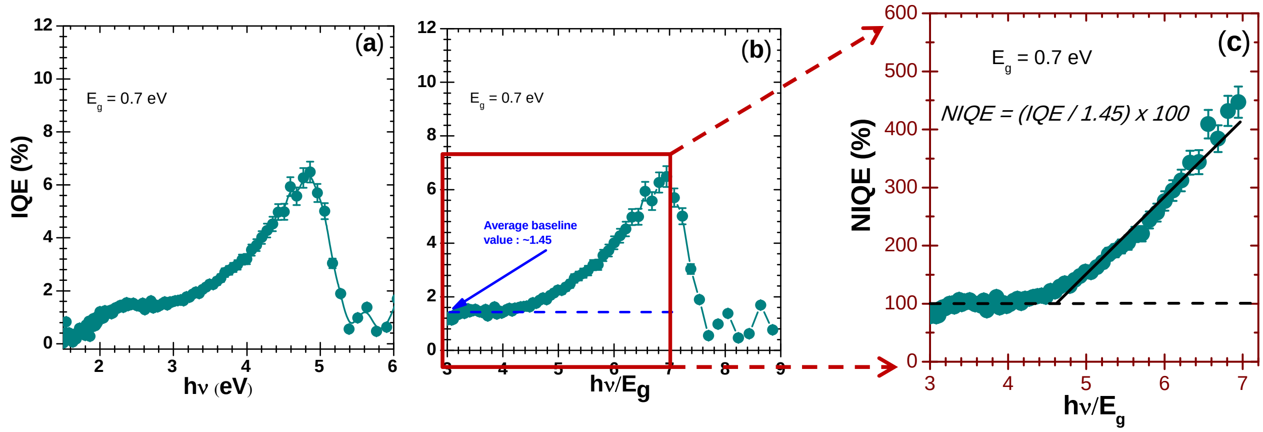

Figure S4: The different processing steps of the IQE spectrum for Eg = 0.7 eV to obtain its corresponding NIQE curve: (**a**) the IQE spectrum as it appears in Fig.5b of the MS; (b) the same IQE spectrum replotted against hν/E_g, while identifying the average IQE value for the baseline and locating the region of interest (Red square) for NIQE calculations; (c) the final corresponding NIQE curve obtained after normalizing with the average baseline value.

References

1. Sukhovatkin, V., Hinds, S., Brzozowski, L. & Sargent, E. H. Colloidal quantum-dot photodetectors exploiting multiexciton generation. *Science* 324, 1542-1544 (2009).
2. Semonin, O. E. *et al*. Peak external photocurrent quantum efficiency exceeding 100% via MEG in a quantum dot solar cell. *Science* 334, 1530-1533 (2011).
3. Midgett, A. G. *et al*. Size and composition dependent multiple exciton generation efficiency in PbS, PbSe, and PbSxSe1−x alloyed quantum dots. *Nano Lett*. 13, 3078-3085 (2013).
4. Aerts, M. *et al*. Highly efficient carrier multiplication in PbS nanosheets. *Nat. Commun*. 5, 3789 (2014).
5. Kim, S. J., Kim, W. j., Sahoo, Y., Cartwright, A. N., Prasad, P.N. Multiple exciton generation and electrical extraction from a PbSe quantum dot photoconductor. Appl. Phys. Lett. 92, 031107 (2008).
